# American Medical Association

**Published:** 1884-07

**Authors:** 


### AMERICAN MEDICAL ASSOCIATION.

At the meeting of the American Medical Association held at Washington in May last, an Amendment to Regulation II. was adopted, which provides that—

Membership in the Association shall be obtainable by any member of a State or County Medical Society recognized by the Association, upon application indorsed by the President and Secretary of said Society; and shall be retained so long as he shall remain in good standing in his local Society, and shall pay his annual dues to the Association.

Applications for membership, in the manner specified above, accompanied with FIVE DOLLARS for annual dues, should be sent directly to the Treasurer, Dr. Richard J. Dunglison, Lock Box 1274, Philadelphia, Pa.; on receipt of which the weekly Journal of the Association will be forwarded for one year to such member.

